# Long-read sequencing reveals two chromosomes and one plasmid in *Vibrio* sp. strain MAO6 isolated from a Marine Beach in Fukui, Japan

**DOI:** 10.1128/mra.00369-24

**Published:** 2024-07-15

**Authors:** Mao Tsukamoto, Yuka Machii, Takafumi Kataoka, Ryuji Kondo

**Affiliations:** 1 Department of Marine Science and Technology, Fukui Prefectural University, Obama, Fukui, Japan; Department of Biological Sciences, Wellesley College, Wellesley, Massachusetts, USA

**Keywords:** *Vibrio*, two chromosomes, plasmids

## Abstract

A marine bacterium *Vibrio* sp. strain MAO6 was isolated from a marine beach in Fukui, Japan. Its complete genome comprises two circular chromosomes (3,418,677 bp and 1,629,627 bp) and one circular plasmid (299,686 bp).

## ANNOUNCEMENT

The genus *Vibrio* is a large group of ubiquitous aquatic bacteria and is mainly isolated from marine environments (1). In the course in microbiology as part of first-year university education at the Department of Marine Science and Technology, Fukui Prefectural University, a marine bacterium *Vibrio* sp. strain MAO6 developed on 1/50-diluted Marine Agar 2216 (BD Difco) was isolated from surface water in the Ano swimming area in Obama, Fukui, Japan (35°32′02.6″N 135°47′12.5″E) on 22 July 2023. A BLAST search (2) for the partial 16S rRNA gene sequence of strain MAO6, obtained by direct sequencing of PCR amplicon using bacterial universal primers 314f (5′-CCTACGGGNGGCWGCAG-3′) (3) and 907r (5′-CCGTCAATTC MTTTGAGTTT-3′) (4), returned *Vibrio agarivorans* strain 289^T^ (NR_028946.1) with a sequence identity of 97.88%. This suggested that strain MAO6 represented a novel species of the genus *Vibrio* according to species delineation using 16S rRNA gene sequence (5). The genome sequence of strain MAO6 is an important resource to define the relatedness within species of the genus *Vibrio*.

Strain MAO6 stored at −80°C with 25% (vol/vol) glycerol was inoculated into Marine Broth 2216 (BD Difco) and incubated for 3 days at 20°C in the dark. Then, the cell pellet was harvested by centrifugation at 3,310 × *g* and 4°C for 30 min. The standard phenol-chloroform extraction method was used to extract genomic DNA (gDNA) from the cell pellet, as described previously (6). After the final purification of the extracted DNA using a short-read eliminator kit (Circulomics), the gDNA was sheared to 10- to 20-kb fragments using g-TUBE device (Covaris). A long-read DNA library was constructed for strain MAO6 using the SMRTbell Express template prep kit v2.0 (PacBio) and sequenced using the PacBio Sequel IIe system. High-fidelity (Hi-Fi) reads were obtained from the subreads generated using circular consensus sequencing via SMRT Link v10.1.00.119528. The 27,182 Hi-Fi reads containing >3,000 bp were screened using Filtlong v0.2.1 (https://github.com/rrwick/Filtlong), and the remaining 22,419 reads were used for *de novo* assembly performed with the Flye v2.9.2 (7) with read-error of 0.01, minimum overlap length between reads of 1,000, and assembly type of meta. Default parameters were used for all software unless otherwise specified. This resulted in three circular genomes with a 42 × coverage (*N*_50_ = 3,418,677 bp). The genomes were annotated and rotated using DFAST (8) available at the DNA Data Bank of Japan (DDBJ).

The complete genome sequence of *Vibrio* sp. strain MAO6 consists of three replicons, including two circular chromosomes and a circular plasmid pMAO6 (Table 1). Within the large chromosome 1, one rRNA operon contained two 5S rRNA genes and two tandem triplications and a single duplication of the rRNA operons were found. Twelve 16S rRNA gene sequences were different with sequence identities from 95.3% to 99.5% to each other. The 16S rRNA gene copy variants in strain MAO6 appears to be at levels of interspecies variation of bacteria according to species delineation using 16S rRNA gene sequence (5).

**TABLE 1:** Genomic features of *Vibrio* sp. strain MAO6

Parameter	Chromosome 1	Chromosome 2	Plasmid pMAO6
Total size (bp)	3,418,677	1,629,627	299,686
Coverage (x)	42	40	49
G + C content (%)	48.6	47.0	44.5
No. of coding DNA sequences	3,043	1,368	314
No. of rRNA	37	0	0
No. of tRNA	117	9	0

## Data Availability

The sequences of large and small chromosomes and plasmid of the MAO6 have been deposited in DDBJ under the accession numbers AP029599, AP029600, and AP029601, respectively. The raw reads have been deposited in the DDBJ Sequence Read Archive under accession number DRR530219. The BioProject and BioSample accession numbers are PRJDB17504 and SAMD00746872, respectively.
